# Starch Synthesis in *Ostreococcus tauri*: The Starch-Binding Domains of Starch Synthase III-B Are Essential for Catalytic Activity

**DOI:** 10.3389/fpls.2018.01541

**Published:** 2018-10-25

**Authors:** Julieta Barchiesi, Maria Belen Velazquez, Nicolas Palopoli, Alberto A. Iglesias, Diego F. Gomez-Casati, Miguel Angel Ballicora, Maria Victoria Busi

**Affiliations:** ^1^Centro de Estudios Fotosintéticos y Bioquímicos (CEFOBI-CONICET), Universidad Nacional de Rosario, Rosario, Argentina; ^2^Departamento de Ciencia y Tecnología, Universidad Nacional de Quilmes and CONICET, Bernal, Argentina; ^3^Laboratorio de Enzimología Molecular, Instituto de Agrobiotecnología del Litoral (CONICET- Universidad Nacional del Litoral) and Facultad de Bioquímica y Ciencias Biológicas, Santa Fe, Argentina; ^4^Department of Chemistry and Biochemistry, Loyola University Chicago, Chicago, IL, United States

**Keywords:** starch, starch synthase, starch-binding domains, microalgae, enzyme regulation

## Abstract

Starch is the major energy storage carbohydrate in photosynthetic eukaryotes. Several enzymes are involved in building highly organized semi-crystalline starch granules, including starch-synthase III (SSIII), which is widely conserved in photosynthetic organisms. This enzyme catalyzes the extension of the α-1,4 glucan chain and plays a regulatory role in the synthesis of starch. Interestingly, unlike most plants, the unicellular green alga *Ostreococcus tauri* has three SSIII isoforms. In the present study, we describe the structure and function of OsttaSSIII-B, which has a similar modular organization to SSIII in higher plants, comprising three putative starch-binding domains (SBDs) at the N-terminal region and a C-terminal catalytic domain (CD). Purified recombinant OsttaSSIII-B displayed a high affinity toward branched polysaccharides such as glycogen and amylopectin, and to ADP-glucose. Lower catalytic activity was detected for the CD lacking the associated SBDs, suggesting that they are necessary for enzyme function. Moreover, analysis of enzyme kinetic and polysaccharide-binding parameters of site-directed mutants with modified conserved aromatic amino acid residues W122, Y124, F138, Y147, W279, and W304, belonging to the SBDs, revealed their importance for polysaccharide binding and SS activity. Our results suggest that *OT_ostta13g01200* encodes a functional SSIII comprising three SBD domains that are critical for enzyme function.

## Introduction

Starch plays a key role in the life cycle of plants as the primary form of carbohydrate storage for chemical energy. Additionally, it has acquired immense value as a vital source of energy and as a precursor of new biodegradable materials (Ball and Morell, 2003; Sticklen, 2006). Starch accumulates as semicrystalline granules with a complex structure and organization composed of two polymers, amylose (AM) and amylopectin (AP). In both polymers, glucose is connected by α-1,4 glycosidic bonds, forming linear chains, and α-1,6 bonds in the so-called branch points. AM is a mostly linear polymer, accounting for between 15 and 35% of the weight of granules. AP has a larger number of branches (5%) and is the dominant component, accounting for 65–85% of granules (Nakamura, 2002; Zeeman et al., 2010; Pfister and Zeeman, 2016).

There are four biochemical steps in starch synthesis (substrate activation, chain elongation, chain branching, and chain debranching) carried out by different enzymes (Preiss et al., 1991; Zeeman et al., 2010). In green plants, soluble and granule-bound starch synthases (SS and GBSS, respectively), which mainly utilize ADP-glucose (ADPGlc), are derived from the bacterial symbiont that gave rise to each branch of the Archeplastida. By contrast, SSs from red algae and glaucophytes mainly utilize UDP-glucose (UDPGlc) and are soluble forms derived from the host, while GBSS-like proteins are derived from the symbiont (Patron and Keeling, 2005; Deschamps et al., 2008a,b,c).

The first isolation of *Ostreococcus tauri* dates from 1994 in a lagoon in Thau, France. This picoalga is a member of the Prasinophyceae, which places it in a key phylogenetic position as a study model because it shares the common ancestor of Chorophyta that currently dominate terrestrial photosynthesis. *O. tauri* lacks a cell wall and flagella, and contains a large nucleus, a single chloroplast, a mitochondrion, and a Golgi apparatus in a reduced cytoplasmic compartment (Demir-Hilton et al., 2011). Although *O. tauri* constitutes the smallest eukaryotic cell and possesses the smallest genome of a eukaryotic photosynthetic organism described to date, it contains more SSIII-like genes (three copies) than any other algae or plant species (e.g., *Arabidopsis thaliana* has only one copy) (Deschamps et al., 2008c). Thus, the genome of *O. tauri* encodes three SSIIIs (SSIII-A, SSIII-B, and SSIII-C) of which only SSIII-C has been characterized (Barchiesi et al., 2017). Interestingly, the *O. tauri* genome lacks genes related to yeast or mammalian glycogenin. Thus, the conservation throughout evolution of the three SSIII isoforms, and the absence of SSIV, could be related to the presence of a single starch granule in this alga with a particular partitioning and propagation mechanism (Ral et al., 2004; Deschamps et al., 2008c).

It is important to note that SSs in both plants and algae have a modular organization. The C-terminal region of all SS isoforms includes a conserved glycosyltransferase (GT) domain, while the N-terminal region includes starch-binding domains (SBD), a specific group of carbohydrate-binding modules (CBM). The generic term CBM refers to a contiguous amino acid sequence with a distinctive fold within an enzyme that may or may not be catalytically active with carbohydrate substrates (Penninga et al., 1996; Boraston et al., 2004; Guillén et al., 2010). The ability of CBMs to bind polysaccharides can be attributed, at least partially, to several aromatic residues that are assembled on a hydrophobic surface. Specifically, SBDs have gained the evolutionary advantage of enabling CBMs to disrupt the structure of the substrate more efficiently due to the presence of two polysaccharide-binding sites (Tormo et al., 1996; Southall et al., 1999). The SSIII isoform of *A. thaliana* (ArathSSIII), containing a GT5 domain^1^ at its C-terminal end and three *in tandem* SBDs belonging to the CBM53 family in its N-terminal region (designated D1-D2-D3), demonstrated the importance and functionality of an SBD in a biosynthetic enzyme (Palopoli et al., 2006; Busi et al., 2008; Valdez et al., 2008, 2011). The CBM53 family currently includes 96 entries in the CAZy database (see footnote 1), and it is widely distributed in archaea, bacteria, and eukaryotes. Three-dimensional structures of numerous CBMs are available for nine families, but structures of CBM45 and CBM53 families have not yet been reported.

Herein, we describe the cloning and expression of the OsttaSSIII-B gene, and purification and characterization of the recombinant protein produced in *Escherichia coli*. Our results show that the OsttaSSIII-B isoform is a modular protein with three N-terminal SBDs and a C-terminal CD, being the SBDs important for its catalytic activity. The purified recombinant protein displayed a high affinity toward branched polysaccharides such as glycogen and AP. Analysis of enzyme kinetic and polysaccharide-binding parameters of OsttaSSIII-B site-directed mutants with altered conserved aromatic amino acid residues W122, Y124, F138, Y147, W279, and W304, belonging to the SBDs, revealed their importance for polysaccharide binding and SS activity.

## Materials and Methods

### Strains and Culture Media

*Escherichia coli* strains XL1 Blue (*endA*1, *gyrA*46, *hsdR*17, *lac*^−^, *rec*A1, *rel*A1, *supE*44, *thi-*1, F′ [*proAB*^+^, *lacI*^q^ *lacZ*Δ*M15*, Tn*10*(*tet*^r^)]) and BL21 (DE3) pLysS (F- *ompT gal dcm hsdS*_B_ [r_B_^−^ m_B_^−^] λ[DE3] pLysS [Cm^r^]) were purchased from Stratagene (La Jolla, CA, United States). Bacterial cells were grown at 37°C in Luria Bertani (LB) medium containing the appropriate antibiotics.

### Protein Sequence Analysis

Domains of OsttaSSIII-B were predicted using the KEGG Sequence Similarity Database (SSDB) Motif Search server (Kanehisa et al., 2017). Alignment of OsttaSSIII-B, OsttaSSIII-C, ArathSSIII-CD, AgrtuGS, and EsccoGS amino acids sequences was performed using the ClustalW algorithm of Unipro UGENE v.1.10.4 (Okonechnikov et al., 2012) with default parameters.

### Protein Structure Modeling

Structural models of the D1, D2, and D3 domains were described previously (Barchiesi et al., 2015). These were built by comparative modeling using Modeller v9.13 (Sali, 1995) with templates CBM25-1 of beta/alpha-amylase from *Paenibacillus polymyxa* (PDB code: 2LAA), CBM25 from *Bacillus halodurans* amylase (PDB code: 2C3V), and the glycogen-binding domain of the AMP-activated protein from *Rattus norvegicus* (PDB code: 1Z0N), respectively (Kawazu et al., 1987; Polekhina et al., 2005; Boraston et al., 2006).

### Cloning, Expression, and Purification of OsttaSSIII-B and OsttaSSIII-B CD

The OsttaSSIII-B gene (*OT_ostta13g01200*) was cloned from *O. tauri* genomic DNA (kindly provide by Dr. Evelyne Derelle) into *Kpn*II and *Hind*III restriction enzyme sites of the pRSET-C vector (Invitrogen, CA, United States) using standard molecular biology procedures and primers OtSSIII-B Nt Fwd (TGCGGTACCCGCGTCGGTGGAG) and OtSSIII-B Ct Rev (CTAAGCTTTTACCGACCGATCATC). OsttaSSIII-B CD was amplified from pRSET-C::OsttaSSIII-B using primers OtSSIII-B CD Fwd (AAGGTACCTCTTCACGTCTGTCAC) and OtSSIII-B Ct Rev, and cloned into *Kpn*II and *Hind*III sites of pRSET-C (*Kpn*I and *Hind*III sites are underlined). The expression vectors pRSET-C::OsttaSSIII-B and pRSET-C::OsttaSSIII-B CD (both containing an N-terminal His-tag sequence) were used to transform *E. coli* BL21 (DE3) pLysS, and cells were grown at 37°C for 3 h, then induced with 0.5 mM isopropyl β-*D*-1-thiogalactopyranoside (IPTG) and incubated at 20°C for at least 18 h. Cells were harvested by centrifugation at 5000 × *g* for 15 min at 4°C, and the pellet was resuspended in 20 mM TRIS-HCl (pH 7.5). Cells were disrupted by sonication and centrifuged at 12,000 × *g* for 15 min at 4°C. The homogenate was loaded onto a HiTrap chelating HP column (GE Healthcare BioSciences, Uppsala, Sweden) equilibrated with binding buffer (20 mM TRIS-HCl pH 7.5, 20 mM imidazole). The column was washed with 10–15 volumes of binding buffer, and each protein was eluted using a linear gradient of binding buffer and elution buffer (20 mM TRIS-HCl pH 7.5, 20–500 mM imidazole) (Barchiesi et al., 2017). The presence of OsttaSSIII-B (103.2 kDa) or OsttaSSIII-B CD (56.3 kDa) in the eluted fractions was monitored by sodium dodecyl sulfate-polyacrylamide gel electrophoresis (SDS-PAGE) and Western blot analyses. Fractions containing the protein of interest were stored at −40°C after addition of 20% (v/v) glycerol.

### Construction, Expression, and Purification of Site-Directed Mutants

OsttaSSIII-B mutants W122AY124A, F138A, Y147A, W279A, and W304A were obtained using the QuickChange II site-directed mutagenesis kit (Stratagene, La Jolla, CA, United States). The pRSET-C::OsttaSSIII-B vector was used as template for PCR amplification. The following primers (and their complements) were used (base substitutions in italics and underlined): OtB W122AY124A, GGTGAAGGGGACGTCG*GC*GGTG*GC*CGCCGATGTGAACGTG; OtB F138A, CGCGCGTTCTCGATGGAT*GC*CGTGTTCAAGGGAGA; OtB Y147A, GAGACGGCGAGGCG*GC*CGAGAAGGACGAAG; OtB W279A, AGAACAACGACTGG*GC*GGTCGCCGACGTCC, and OtB W304A, CGTCGA AGAGTCGGCC*GC*GGATAACAACGAACAG. Mutations were confirmed by DNA sequencing (Macrogen, Korea). The resultant plasmids, named pRSET-C::OsttaSSIII-B W122AY124A, pRSET-C::OsttaSSIII-B F138A, pRSET-C::OsttaSSIII-B Y147A, pRSET-C::OsttaSSIII-B W279A, and pRSET-C::OsttaSSIII-B W304A (all containing an N-terminal His-tag sequence) were used to transform *E. coli* BL21 (DE3) pLysS cells. Mutated recombinant proteins were expressed, purified, and stored at −40°C as described above.

### Determination of Denaturation Temperature as an Indicator of Protein Stability

Differential scanning fluorimetry (DSF) was performed on an Applied Biosystems real-time PCR instrument (Giuliani et al., 2008). The technique is based on the properties of some dyes that interact with proteins during thermal unfolding, resulting in an increase in fluorescence upon contact with the hydrophobic core of the protein. Thus, 4 μg of wild-type (WT) or mutated protein was 5000-fold diluted with SYPRO Orange dye to a total volume of 25 μl in 0.2 ml optical 8-Cap Tube Strips (Life Technologies). Samples were heated from 15°C to 95°C with increments of 1°C/min (∼1.5 h) and fluorescence data were collected using a 550 nm filter in melting temperature (Tm) mode. The melting temperature was determined by calculating the first derivative from the melting curve using GraphPad Prism 5 (GraphPad Software, La Jolla, CA, United States). All measurements were performed in duplicate.

### Starch Synthase Activity Assays

The activity of WT and mutated OsttaSSIII-B proteins was determined following the formation of inorganic phosphate (Pi) after hydrolysis of generated ADP by alkaline phosphatase by colorimetric assays as previously reported (Barchiesi et al., 2017). The reaction medium (50 μl final volume) contained 50 mM BICINE pH 8.0, 2 U of *E. coli* alkaline phosphatase (Sigma-Aldrich, MO, United States), 0–1 mM ADPGlc and one of the following polysaccharides: 0–2 mg/ml rabbit liver glycogen (G8876 Sigma-Aldrich), 0–3 mg/ml potato AP (10118; Fluka), 0–1 mg/ml potato AM (10130; Fluka), or 0–2 mg/ml potato starch (85649; Fluka). Reaction vessels were gently shaken all over the assay to keep substrates in suspension. When UDPGlc was used as the glycosyl donor, all other assay conditions were identical, but ADPGlc was replaced with UDPGlc in the range of 0–10 mM. After incubation for 20 min at 30°C, the reaction was stopped by the addition of Malachite Green–ammonium molybdate (MG–am) reagent. This MG–am solution was prepared by merging three volumes of 0.5 mM aqueous solution of Malachite Green (oxalate salt from Sigma, M6880) and one volume of 0.034 M ammonium molybdate in 4 M HCl. The color reaction was developed by mixing samples (1.0 volume) with the color reagent (6.7 volumes). The complex formed from the released Pi was measured at 630 nm as described (Fusari et al., 2006).

Enzyme kinetic data were plotted as specific activity (μmol/min/mg protein) versus substrate concentration. All kinetic parameters [*V*_max_, *S*_0.5_ and Hill number (*n*_H_)] were determined from a non-linear fit of the Michaelis–Menten equation to the observed enzyme activities using GraphPad Prism v.6.01. The average values of at least three determinations ± SE are reported. Total protein concentration was determined using the Bradford method (Bradford, 1976).

A pH profile of OsttaSSIII-B activity was obtained by measuring activity at 30°C and pH values ranging from 6.0 to 10.0 in 50 mM BICINE buffer. The temperature profile of OsttaSSIII-B activity was followed by measuring activity at pH 8.0 and temperature values ranging from 20 to 45°C in 50 mM BICINE buffer. Both experiments were run with 2 mg/ml glycogen and 0.5 mM ADPGlc. Standard kinetic parameters were determined at pH 8.0 and 30°C.

### Polysaccharide-Binding Assays

The adsorption constant (*K*_ad_) was measured as described previously with minor modifications (Valdez et al., 2008; Wayllace et al., 2010; Barchiesi et al., 2015). Purified recombinant proteins (final concentration of 0–80 μM) were mixed with starch, AM, or AP in 20 mM TRIS-HCl (pH 7.5) at a final polysaccharide concentration of 10% (w/v) in a final volume of 60 μl. A control without polysaccharide was included. Binding was performed at 4°C for 8 min with gentle shaking (10 rpm) and centrifuged at 12,000 × *g* for 1 min. To calculate the amount of bound protein, the total protein present in the pellet of the control reaction was subtracted from the amount of protein obtained in the pellet of the reactions with each polysaccharide. Protein concentration was determined by Bradford method (Bradford, 1976), and the adsorption constant (*K*_ad_, in ml per g of polysaccharide) was determined from the slope as previously reported (Valdez et al., 2008; Wayllace et al., 2010; Barchiesi et al., 2015).

## Results and Discussion

### Predicted Polysaccharide-Binding Residues and Conserved Structural Features of OsttaSSIII-B

**FIGURE 1:**
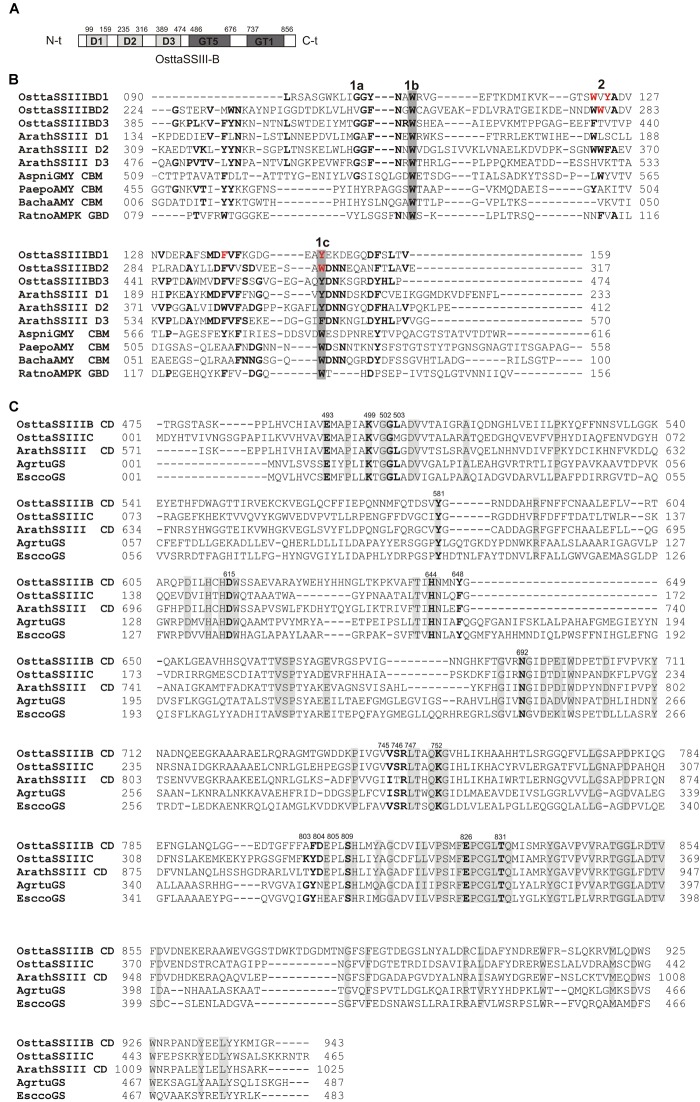
**(A)** Schematic representation of OsttaSSIII-B. D1, D2, D3, GT5, and GT1 domains are shown. **(B)** ClustalW alignment of OsttaSSIII-B D1, D2, and D3, ArathSSIII (*Arabidopsis thaliana* SSIII) D1, D2, and D3, AspniGMY (*A. niger* glucoamilase) CBM20, and RatnoAMPK β-subunit GBD (CBM48), BachaAMY CBM25, and PaepoAMY CBM25, used as templates for starch-binding domain (SBD) homology modeling. 1 and 2 above the alignment indicate binding sites 1 and 2 characterized in *A. thaliana*. 1a, 1b, and 1c indicate G335, W340, and Y394, and 2 indicates W366 (ArathSSIII numbering). OsttaSSIII-B amino acids residues W122, Y124, F138, Y147, W279, and W304 are indicated in red. Other previously characterized binding residues are shown in bold. Gray boxes indicate conserved residues. **(C)** ClustalW alignment of OsttaSSIII-B, OsttaSSIII-C, ArathSSIII-CD (*Arabidopsis thaliana* SSIII catalytic domain), AgrtuGS (*Agrobacterium tumefaciens* glycogen synthase), and EsccoGS (*E. coli* glycogen synthase). Previously characterized catalytic residues are shown in bold. Gray boxes indicate identical residues.

We previously reported that *O. tauri* possesses three SSIII isoforms. Analysis of the OsttaSSIII-B amino acid sequence using the SSDB Motif Search server (Sato, 2001) revealed that this protein contains three N-terminal SBDs (*E*-values = 9.5e^−6^, 2.7e^−12^, and 3.9e^−15^, respectively; Figure 1A). Our results indicate that the domain organization of OsttaSSIII-B is most similar to the SSIII from higher plants than the other two OsttaSSIII isoforms. Figure 1B shows the amino acid sequences alignment between SBDs from OsttaSSIII-B with the following previously characterized SBDs: D1, D2, and D3 N-terminal type SBDs from ArathSSIII; a C-terminal type SBD from *Aspergillus niger* glucoamylase (Genbank: CAK38411.1); the glycogen-binding domain (GBD) of an AMP-activated protein from *R. norvegicus*; CBM25 from *B. halodurans* amylase; and CBM25 beta/alpha-amylase from *P. polymyxa*. These structures were used as templates for homology modeling. The starch binding sites described for ArathSSIII D2 are highly conserved in OsttaSSIII-B SBDs, specifically residues G335, W340, and Y394 (ArathSSIII numbering) belonging to binding site 1 (Valdez et al., 2011; Barchiesi et al., 2015). By contrast, binding site 2, which is essential for binding to starch, AM, and AP, is only conserved in OsttaSSIII-B D2 (W279, OsttaSSIII-B numbering). However, the neighboring residue W365 is conserved in OsttaSSIII-B D1 (W122, OsttaSSIII-B numbering), and another aromatic amino residue is present at position 124 (Y124).

Analysis of the OsttaSSIII-B catalytic domain (CD) amino acid sequence was also performed with the SSDB Motif Search server. We found that half of the CD has a structure similar to the GT5 family (Pfam 343 PF08323), while the other half has a structure similar to GT1 (Pfam PF00534), with relatively low *E*-values (9e^−42^ and 2.4e^−9^, respectively; Figure 1A). Alignment of OsttaSSIII-B CD, OsttaSSIII-C, ArathSSIII-CD, EsccoGS, and AgrtuGS amino acid sequences revealed high conservation of residues involved in substrates binding and catalysis in the *O. tauri* SSIII-B isoform (Figure 1C and Tables 1, 2).

**Table 1:** Amino acid residues involved in glycogen binding in ArathSSIII CD, EsccoGS, and OsttaSSIII-B CD.

EsccoGS	OsttaSSIII-B	ArathSSIII	Function
Glu9	Glu493	Glu585	Binding of Glc + 1
Leu19	Leu503	Leu595	Binding of Glc + 1 (KTGGL/KVGGM)
Tyr 95	Tyr581	Tyr672	Stacking With Glc + 2
Asp137	Asp615	Asp706	H-bond with OH_2_ and OH_3_ Glc + 1
Tyr165	Tyr648	Phe740	Stacking with Glc + 2

*Ref: For the Arabidopsis thaliana SSIII isoform, Glc + 1 and Glc + 2 indicate which Glc in glycogen is involved in the interaction; OH2 and OH3 indicate the OH groups of Glc + 1 that interact with the protein; KTGGL and KVGGM correspond to the conserved motifs in *E. coli* GS and OsttaSSIII-B enzymes, respectively.*

**Table 2:** Amino acid residues involved in ADPGlc binding in ArathSSIII, EsccoGS, and OsttaSSIII-B.

EsccoGS	OsttaSSIII-B	ArathSSIII	Function
Lys15	Lys499	Lys591	ADPGlc binding (KTGGM/KVGGL)
Gly18	Gly502	Gly594	ADPGlc binding (KTGGM/KVGGL)
His161	His644	His736	H-bond with O_6_ ribose (380 loop)
Asn246	Asn692	Asn783	H-bond with O_6_ ribose
Val297	Val745	Ile835	H-bond with O_2_ ribose
Ser298	Ser746	Thr836	vdW with adenine
Arg300	Arg747	Arg837	Ionic interaction with Pi
Lys305	Lys752	Lys842	Ionic interaction with Pi
Gly354	Ala803	Thr896	Interaction with adenine
Tyr355	Phe804	Tyr897	Stacking with adenine
His356	Asp805	Asp898	Carbonyl interacts with adenine
Ser360	Ser809	Ser902	vdW with adenine
Glu377	Glu826	Glu919	Ionic interaction with Pi
Thr382	Thr831	Thr924	H-bond with O_2_ ribose

*Ref: AthSSIII, Arabidopsis thaliana SSIII isoform; Adenine, ribose and phosphate (Pi) indicate which part of the ADP-Glc participates in each interaction; O2 and O6, oxygen atoms in glucose; vdW, van der Waals interactions; KTGGM and KVGGL correspond to the conserved motifs in E. coli GS and OsttaSSIII-B enzymes, respectively.*

**FIGURE 2:**
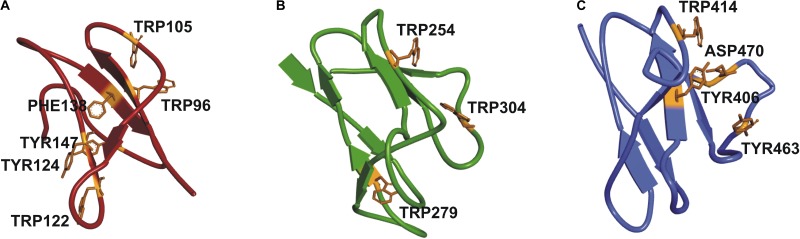
Homology modeling of OsttaSSIII-B SBDs **(A)** D1 (red); **(B)** D2 (green), and **(C)** D3 (blue) domains, respectively, of OsttaSSIII-B, showing the residues involved in binding in orange.

It was not possible to construct a homology model of the full-length protein due to lack of a crystal structure of a protein with the same structural organization as SSIII, with three N-terminal SBDs and one C-terminal CD. Instead, we have built homology models of the individual SBDs (Barchiesi et al., 2015) (see Figure 2). Despite the overall architecture of each SBD being quite similar, differences in amino acid residues in the ligand-binding sites, and the relative distances between them and the catalytic domain may impose different specificities for portions of a starch molecule. These observations motivated us to probe the functional importance of this organization by comparing the activity of WT OsttaSSIII-B with mutants harboring altered starch binding sites, and a truncated OsttaSSIII-B CD variant lacking the SBDs.

### Expression and Purification of WT and Mutated OsttaSSIII-B Proteins

**FIGURE 3:**
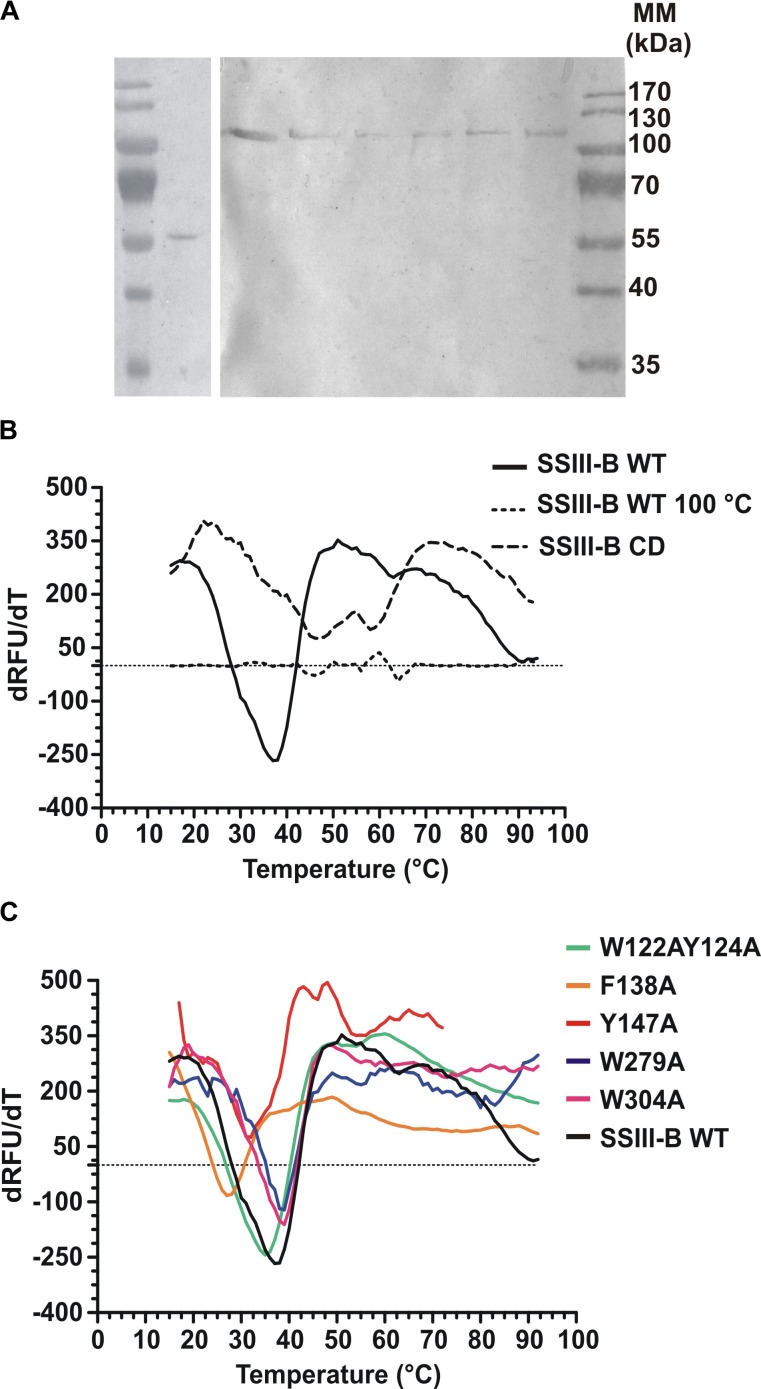
**(A)** Western blot of recombinant enzymes. Lane 1, Molecular weight standards; lane 2, OsttaSSIII-B CD; lane 3, OsttaSSIII-B WT; lane 4, OsttaSSIII-B W122AY124A; lane 5, OsttaSSIII-B F138A; lane 6, OsttaSSIII-B Y147A; lane 7, OsttaSSIII-B W279A; lane 8, OsttaSSIII-B W304A. Numerals indicate molecular masses of the prestained SDS-PAGE standards (Low Range, BioRad). **(B,C)** First derivatives [-d(RFU)/dT] from the melting curves determined using differential scanning fluorimetry. Melting temperature (Tm) was obtained from the *x*-axis value (T) that corresponds with the lowest *y*-axis value of the curve. SSIII-B 100°C is the curve corresponding to the wild-type (WT) protein previously denatured by heating for 15 min at 100°C, used as an unfolded protein control.

DNA fragments encoding full-length OsttaSSIII-B and truncated OsttaSSIII-B CD were cloned into the pRSET-C vector for expression of proteins with N-terminal His-tags. To analyze the functions of the aromatic residues W122, Y124, F138, Y147, W279, and W304 in OsttaSSIII-B polysaccharide binding and enzymatic activity, the modified proteins OsttaSSIII-B W122AY124A, F138A, Y147A, W279A, and W304A were obtained by site-directed mutagenesis. Western blot assays revealed that the size of the mutated proteins was comparable to that observed for the WT enzyme (Figure 3A), with a molecular mass of 103.2 kDa for full-length forms and 56.3 kDa for OsttaSSIII-B CD. Protein melting temperature curves were obtained for WT OsttaSSIII-B, the truncated CD variant, and five modified enzymes, and the melting temperature (Tm) was determined (Figures 3B,C). Fluorescence-based thermal shift assays showed that the melting profiles of mutant proteins were similar to that of the WT enzyme, indicating that overall folding was conserved. Nevertheless, the mutation F138A caused a decrease of 10°C in Tm, whereas mutations W122AY124A and Y147 lowered the Tm by 2.5 and 4.5°C, respectively. OsttaSSIII-B CD showed two distinct melting temperatures, both higher than the Tm value for the full-length WT enzyme (46.2 and 57.5°C). This observation may suggest successive melting of independent protein domains caused by the absence of the N-terminal region. From these results, we conclude that the mutated proteins were properly folded, but their thermal stability was somewhat reduced by introduction of mutations in the D1 domain.

### Kinetic Characterization of OsttaSSIII-B

**FIGURE 4:**
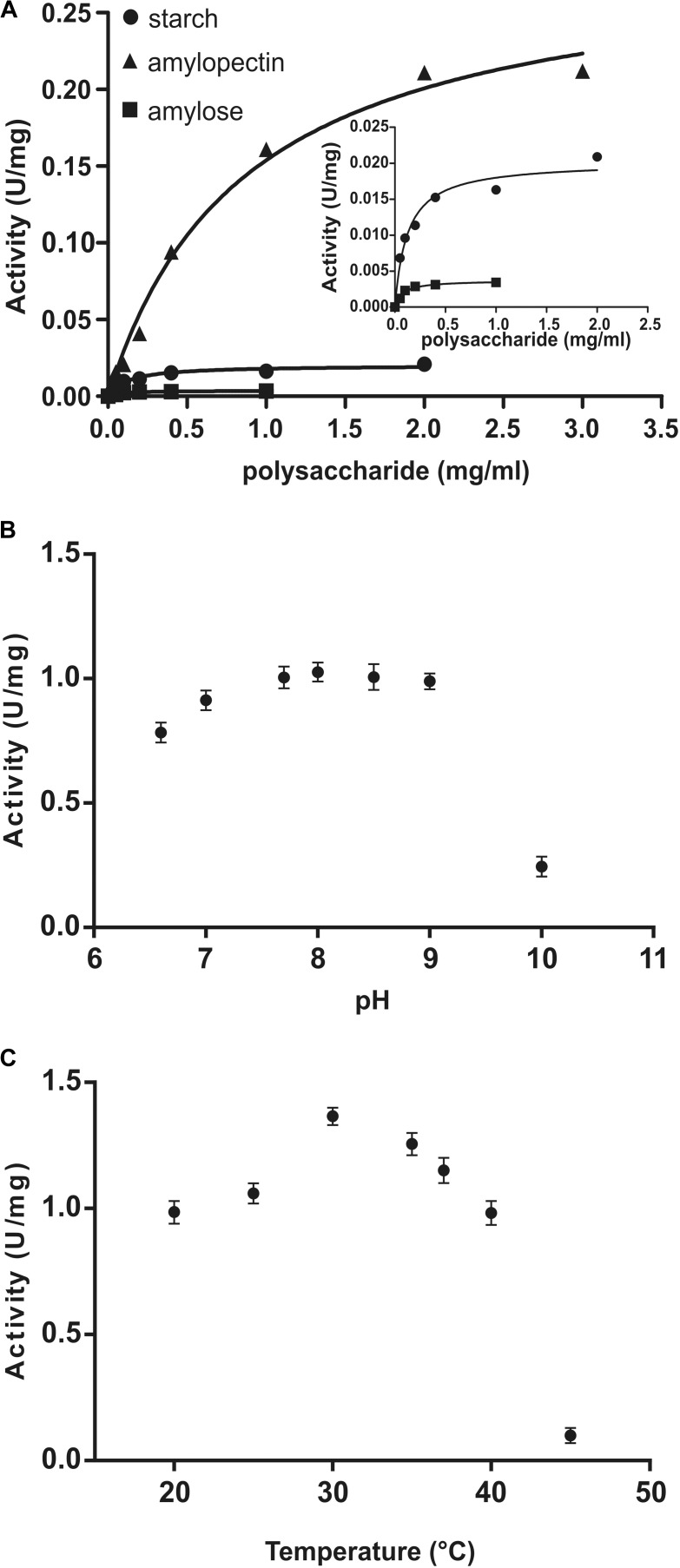
**(A)** Starch (circles), amylose (squares), and amylopectin (triangles) saturation plots for WT OsttaSSIII-B measured in the presence of 500 μM ADPGlc. **(B)** Effect of pH on the activity of WT OsttaSSIII-B. 50 mM BICINE and 50 mM glycine buffers were used for pH 6.0 – 10.0. Assays were run with 2 mg/ml glycogen and 500 μM ADPGlc. **(C)** Effect of temperature on the activity of WT OsttaSSIII-B. Assays were run with 2 mg/ml glycogen and 500 μM ADPGlc, in 50 mM BICINE buffer pH 8, with reaction temperatures of 20, 25, 30, 35, 40, and 45°C.

**FIGURE 5:**
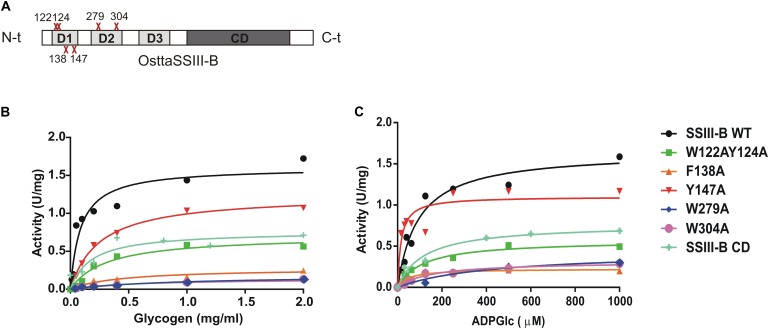
**(A)** Schematic representation of the mutated proteins used in this study. Residues W122, Y124, F138, Y147, W279, and W304 were replaced by alanine to yield OsttaSSIII-B W122AY124A, F138A, Y147A, W279A, and W304A. Amino acid locations are indicated with a red cross. CD, catalytic domain; D1, D2, and D3, individual SBD modules. **(B)** Glycogen and **(C)** ADPGlc saturation plots for WT OsttaSSIII-B and mutated enzymes performed in the presence of 500 μM ADPGlc or 2 mg/ml glycogen, respectively.

Kinetic parameters were calculated for WT OsttaSSIII-B from saturation plots of enzymatic activity using ADPGlc as the sugar-nucleotide donor, and glycogen, starch, AM, or AP as the acceptor substrate (Figures 4A, 5A,B and Table 3). In all conditions, OsttaSSIII-B exhibited typical Michaelis-Menten kinetics. The *S*_0.5_ values for the acceptor polysaccharide were 0.08 mg/ml for glycogen, 0.12 mg/ml for starch, 0.08 mg/ml for AM, and 1.04 mg/ml for AP. By contrast, the *S*_0.5_ value for ADPGlc was 90 μM when glycogen was used as the acceptor substrate (Table 3). The *S*_0.5_ value for glycogen was about 75% lower than those reported for other SSIIIs, such as OsttaSSIII-C and ArathSSIII (*S*_0.5_ = 0.23 and 0.26 mg/ml, respectively) (Valdez et al., 2008; Wayllace et al., 2010; Barchiesi et al., 2017). On the other hand, *n*_H_ values for ADPGlc and glycogen were about 1.16 and 0.98, respectively, whereas for AP and AM were 1.48 and 1.60, respectively, suggesting a cooperative kinetics for these polysaccharides.

**Table 3:** Kinetic parameters of wild-type (WT) OsttaSSIII-B.

	*S*_0.5_ (ADPGlc mM, Polysacc mg/ml)	*n*_H_	*V*_max_ (U/mg)
ADPGlc	0.09 ± 0.02	1.16 ± 0.23	1.65 ± 0.09
Glycogen	0.08 ± 0.02	0.98 ± 0.10	1.61 ± 0.09
Starch	0.12 ± 0.02	0.48 ± 0.20	0.02 ± 0.01
Amylopectin	1.04 ± 0.17	1.48 ± 0.23	0.30 ± 0.02
Amylose	0.08 ± 0.01	1.60 ± 0.22	0.003 ± 0.001

OsttaSSIII-B specific activity was higher with glycogen respect to those obtained for starch, AM, or AP (80-, 537-, and 5-fold higher, respectively). The lower specific activity observed with starch may be due to an inhibitory effect of the amylose present in this polysaccharide. It should be noted that OsttaSSIII-B showed a lower *S*_0.5_ for amylose than for starch or amylopectin (see Table 3).

Similar results, demonstrating highest activity with glycogen as acceptor substrate, were previously reported for ArathSSIII (Valdez et al., 2008) and OsttaSSIII-C (Barchiesi et al., 2017). OsttaSSIII-B did not exhibit enzymatic activity under the assayed conditions when UDPGlc was used as the donor substrate. Furthermore, OsttaSSIII-B displayed an optimum reaction temperature of 30°C, and a pH optimum of 8 (Figures 4B,C), consistent with the alkaline pH of the chloroplast stroma. Based on these results, we further studied the kinetic parameters of OsttaSSIII-B WT, truncated, and modified forms using glycogen as the acceptor polysaccharide and ADPGlc as the glycosyl donor, at pH 8 and 30°C.

### The N-Terminal SBDs Are Important for OsttaSSIII-B Starch Synthase Activity

In order to analyze the role of the SBDs in the function of OsttaSSIII-B, we evaluated the starch synthase activity of the truncated OsttaSSIII-B CD protein (Figures 5B,C and Tables 4, 5). Under the assay conditions, OsttaSSIII CD exhibited a 50% lower *V*_max_ value than that of the full-length enzyme, whereas the *S*_0.5_ value for ADPGlc was similar. The *S*_0.5_ value for the acceptor polysaccharide was twofold higher than the full-length protein (0.18 and 0.08 mg/ml, respectively; Tables 4, 5). These results suggest that the presence of the SBDs correlates with an increase in the apparent affinity for glycogen.

**Table 4:** Kinetic parameters of WT SSIII-B and mutated proteins for glycogen.

Isoform	*S*_0.5_ (mg/ml)	*n*_H_	*V*_max_ (U/mg)	*V*_max_*/S*_0.5_
SSIII-B WT	0.08 ± 0.02	0.98 ± 0.10	1.61 ± 0.09	20.12 ± 0.30
SSIII-B W122A-Y124A (D1)	0.31 ± 0.09	1.80 ± 0.23	0.71 ± 0.06	2.29 ± 0.37
SSIII-B F138A (D1)	0.40 ± 0.06	0.84 ± 0.16	0.28 ± 0.01	0.70 ± 0.18
SSIII-B Y147A (D1)	0.25 ± 0.05	1.02 ± 0.11	1.24 ± 0.07	4.96 ± 0.26
SSIII-B W279A (D2)	0.54 ± 0.08	0.93 ± 0.09	0.14 ± 0.01	0.26 ± 0.22
SSIII-B W304A (D2)	0.89 ± 0.20	0.88 ± 0.10	0.19 ± 0.02	0.21 ± 0.33
SSIII-B CD	0.18 ± 0.10	2.00 ± 1.32	0.78 ± 0.12	4.33 ± 0.71

**Table 5:** Kinetic parameters of SSIII-B WT and mutated proteins with the substrate ADPGlc.

Isoform	*S*_0.5_ (mM)	*n*_H_	*V*_max_ (U/mg)	*V*_max_/*S*_0.5_
SSIII-B WT	0.09 ± 0.02	1.16 ± 0.23	1.65 ± 0.09	18.33 ± 0.27
SSIII-B W122AY124A (D1)	0.11 ± 0.01	0.94 ± 0.13	0.57 ± 0.02	5.18 ± 0.12
SSIII-B F138A (D1)	0.07 ± 0.02	1.06 ± 0.33	0.23 ± 0.02	3.28 ± 0.37
SSIII-B Y147A (D1)	0.07 ± 0.01	0.78 ± 0.18	1.10 ± 0.08	15.71 ± 0.21
SSIII-B W279A (D2)	0.38 ± 0.11	0.88 ± 0.23	0.42 ± 0.05	1.10 ± 0.41
SSIII-B W304A (D2)	0.15 ± 0.03	0.80 ± 0.16	0.31 ± 0.02	2.06 ± 0.26
SSIII-B CD	0.12 ± 0.04	0.57 ± 0.28	0.76 ± 0.06	6.33 ± 0.4

Amino acid sequence alignment and *homology* modeling revealed the high conservation of specific residues in SBDs potentially implicated in starch binding and catalysis. We previously demonstrated that the OsttaSIII-B D1 and D2 domains share higher sequence identity with ArathSSIII D2 than OsttaSIII-B D3, and that both domains make major contributions to polysaccharide binding. The putative starch-binding residues in the OsttaSIII-B D2 domain, as well as the shorter OsttaSIII-B D1 domain (60 amino acid residues), are highly conserved in ArathSSIII D2 (Barchiesi et al., 2015).

The amino acid residues Y147 and W304 of OsttaSSIII-B align well with Y394 of the ArathSSIII D2 1c binding site, and W279 aligns with W366 of the ArathSSIII D2 binding site 2 (Figure 1A). Additionally, we previously postulated that amino acids W122 and Y124 of OsttaSSIII-B belong to binding site 2 in D1. However, residue F138 is also highly conserved, but this residue has not been characterized before. Therefore, to analyze the functions of these amino acid residues in OsttaSSIII-B activity, kinetic parameters of alanine-substituted mutants were determined as described above in the Materials and Methods section, and the results are shown in Figure 5 and Tables 4, 5. An approximately 50–90% decrease in *V*_max_ was observed for the mutated proteins compared with the WT enzyme, except for the Y147A variant. Using glycogen as the variable substrate, *S*_0.5_ values for the polysaccharide were approximately 3- to 10-fold higher respect to that observed for the WT enzyme (0.31, 0.40, 0.25, 0.54, and 0,89 mg/ml for W122AY124A, F138A, Y147A, W279A, and W304A, respectively) (Table 4 and Figure 5). These results show that there is a remarkable decrease in the apparent affinity for the polysaccharide substrate, while minor changes (until fourfold) in the *S*_0.5_ for ADPGlc were observed (Table 5).

It is important to note that the *S*_0.5_ for glycogen of the CD protein is lower than that of any of the mutated enzymes. While the SBDs present in the full length WT protein increases the affinity for glycogen, the mutated inactive SBDs could block the access of the substrate to the catalytic site, resulting in higher *S*_0.5_ values.

### Effect of SBD Amino Acid Substitutions on OsttaSSIII-B Binding Properties

**FIGURE 6:**
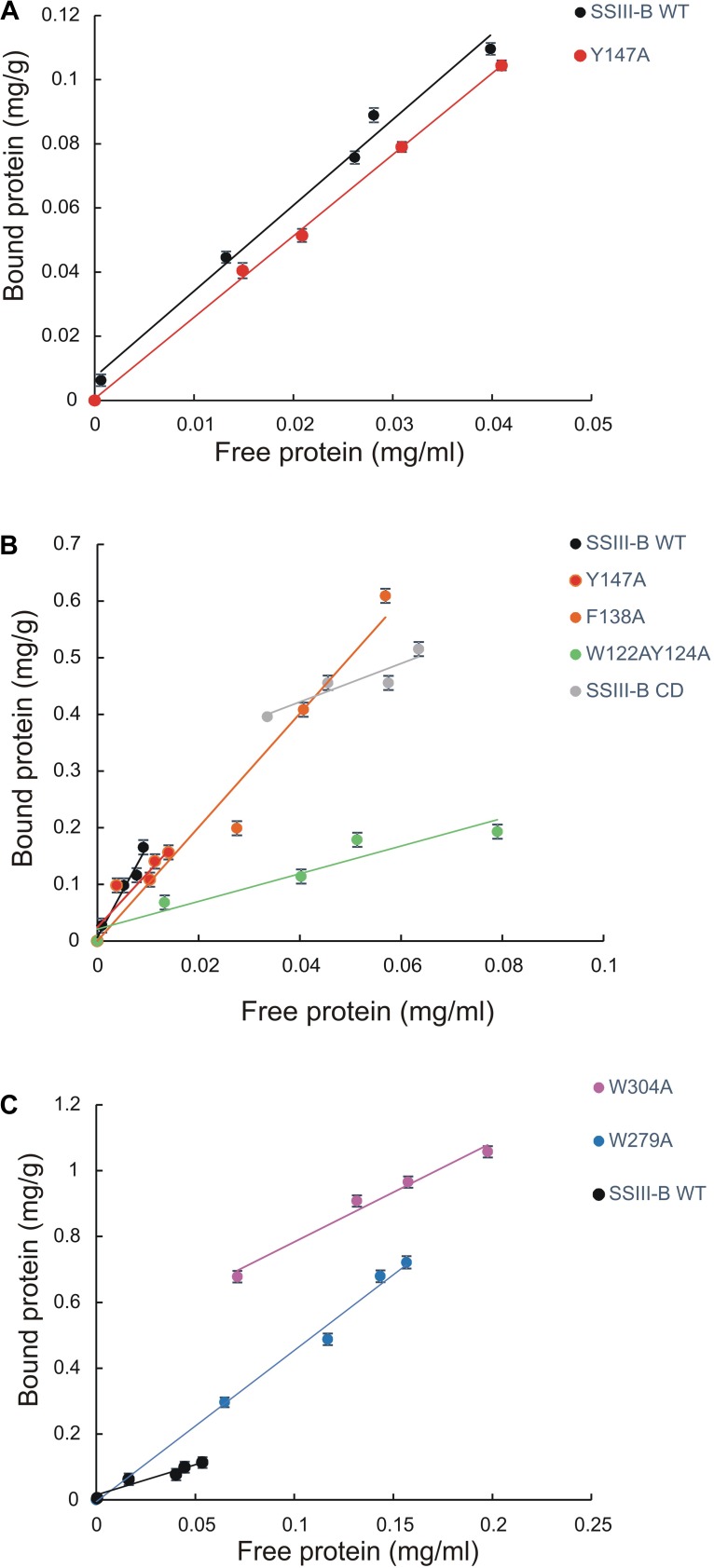
Adsorption of purified OsttaSSIII proteins to polysaccharides. Absorption isotherms are shown with **(A)** starch, **(B)** amylose, **(C)** amylopectin. Linear adsorption isotherms indicate the apparent equilibrium distribution of WT OsttaSSIII-B and mutated proteins between the solid (bound protein) and liquid (free protein) phase at different protein concentrations. Adsorption constants are listed in Table 6 and represent the slopes of the isotherms with units of ml of protein per milligram of polysaccharide (*n* = 2 ± standard error).

We next explored the effect of amino acid substitutions in SBD regions on substrate binding. Figure 6 shows adsorption isotherms for the binding of WT, CD, and mutant OsttaSSIII-B proteins to starch, AM, and AP (see also Table 6). WT OsttaSSIII-B displayed higher affinity for AM (16.61 ml/g) than starch (2.40 ml/g) or AP (1.83 ml/g). These results are consistent with our previous studies on binding using N-terminal SBDs (D123) of WT SSIII-B (*K*_ad_ = 2.22, 7.02, and 3.84 ml/g for starch, AM, and AP, respectively) (Barchiesi et al., 2015), and also with binding of D123 from ArathSSIII (Valdez et al., 2011). The higher binding affinity observed for AM could be due to the insignificant branching degree of this polysaccharide, regards to starch or amylopectin, which could make it more accessible for the large OsttaSSIII-B protein. In the case of the CD, we verified its ability to bind AM (*K*_ad_ = 3.38 ml/g) but the absence of SBDs could cause the lost of the binding capacity to starch and AP (Table 6). Meanwhile, W122AY124A, F138A, and Y147A variants yielded *K*_ad_ values for AM that were 7-, 1.5-, and 1.5-fold lower than the WT enzyme, and all three mutants completely lost their capacity to bind AP, suggesting that these amino acid residues in D1 are essential for binding to the AP component. By contrast, W279A and W304A proteins were unable to bind AM and starch, suggesting both residues are essential for binding to the AM fraction. The Y147A protein was the only isoform capable of binding starch, suggesting that all other studied residues (W122, Y124, F138, W279, and W304) are essential for binding to physiological substrates. Analysis of the spatial position of Y147 in our OsttaSSIII-B model shows that this residue is far away from the other two conserved binding amino acids in D1 (W96 and W105), suggesting that Y147, which aligns with binding site 1c in the D1 domain, is not involved in starch binding, and that D1 likely utilizes another aromatic residue in this position. Interestingly, some OsttaSSIII-B-mutated proteins displayed an increased polysaccharide binding capacity relative to the WT enzyme. The *K*_ad_ values for AP of W279A and W304A variants were 2.6- and 1.5-fold higher than those observed for the WT protein (*K*_ad_ W279A = 4.75 ml/g, and *K*_ad_ W304A = 2.75 ml/g, vs. *K*_ad_ WT = 1.83 ml/g) (Figure 6C and Table 6).

**Table 6:** Adsorption constants for OsttaSSIII-B proteins.

Isoform	*K*_ad_ (ml/g)		
	Starch	Amylose	Amylopectin
OsttaSSIII-B WT	2.40 ± 0.21	16.61 ± 0.91	1.83 ± 0.31
OsttaSSIII-B W122AY124A (D1)	ND	2.44 ± 0.82	ND
OsttaSSIII-B F138A (D1)	ND	11.48 ± 0.61	ND
OsttaSSIII-B Y147A (D1)	2.50 ± 0.43	10.53 ± 0.42	ND
OsttaSSIII-B W279A (D2)	ND	ND	4.75 ± 0.28
OsttaSSIII-B W304A (D2)	ND	ND	2.75 ± 0.26
OsttaSSIII-B CD	ND	3.38 ± 0.73	ND

*ND, No detected.*

Taken together, these results suggest that the AM specificity of the WT enzyme can be attributed to the binding residues in the D2 domain, while the ability to bind AP is related to the D1 domain. However, it is important to emphasize that amino acid residues in both D1 and D2 are essential for binding to starch. This is the first experimental evidence demonstrating the importance of amino acid residues in the D1 domain in the binding capacity of a SS enzyme.

## Conclusion

A better understanding of the properties of CBMs and SBDs and their interactions with catalytic domains could provide new strategies to develop enzymes with improved catalytic efficiency toward polysaccharide substrates. In previous work, we evaluated the effects of mutating several residues in the D2 domain of SSIII from *A. thaliana*. Herein, we analyzed the contribution of several residues from D1 and D2 domains, representing the first characterization of the effects of both domains on the entire protein.

The adsorption parameters of the full-length protein for AM were twice higher than that obtained for the D123 domain alone (Barchiesi et al., 2015), indicating an important contribution of the CD in providing additional binding sites for the linear substrate. Furthermore, mutation of the D1 domain mainly affected AP binding, whereas mutation of D2 mainly affected AM binding, indicating that both SBDs possess different capabilities in the adsorption of different substrates. In addition, mutation of the D2 domain affected the enzyme catalysis, whereas altering the D1 domain had less impact.

Finally, we evaluated the contribution of the two binding sites in the D2 domain, and observed that the W279A mutation moderately decreased the apparent affinity for glycogen, similarly to the W366 mutation in the D2 domain of *A. thaliana* SSIII. However, the W304A mutation (comparable to Y394A in *A. thaliana* SSIII D2) caused a 10-fold decrease in *S*_0.5_ for glycogen, indicating a greater contribution of binding site 1 in the apparent affinity for polysaccharide substrates.

## Author Contributions

JB planned and performed the experiments, analyzed the data, and wrote the manuscript. MBV performed the experiments and analyzed the data. NP performed the structure modeling and analyzed the data. DFG-C, AAI, MAB, and MVB planned the experiments, analyzed the data, and wrote the manuscript.

## Conflict of Interest Statement

The authors declare that the research was conducted in the absence of any commercial or financial relationships that could be construed as a potential conflict of interest.

## Footnotes

1 http://www.cazy.org/
